# Improved depression screening and treatment among low-income pregnant and postpartum women following Medicaid expansion in the U.S

**DOI:** 10.3389/frhs.2022.942476

**Published:** 2022-08-17

**Authors:** Jangho Yoon, S. Marie Harvey, Jeff Luck

**Affiliations:** ^1^Department of Preventive Medicine and Biostatistics, F. Edward Hebert School of Medicine, The Uniformed Services University of the Health Sciences, Bethesda, MD, United States; ^2^School of Social and Behavioral Health Sciences, College of Public Health and Human Sciences, Oregon State University, Corvallis, OR, United States

**Keywords:** Medicaid expansion, depression screening, psychotherapy for depression, antidepressant, pharmacotherapy for depression, postpartum, pregnancy

## Abstract

**Objective:**

This study examined the effect of Medicaid expansion in Oregon under the Affordable Care Act on depression screening and treatment among pregnant and postpartum women who gave Medicaid-financed births.

**Methods:**

Oregon birth certificates were linked to Medicaid enrollment and claims records for 2011–2016. The sample included a policy group of 1,368 women (*n* = 2,831) who gave births covered by pregnancy-only Medicaid in the pre-expansion period (before 2014) and full-scope Medicaid in the post-expansion period, and the comparison group of 2,229 women (*n* = 4,580) who gave births covered by full-scope Medicaid in both pre- and post-expansion periods. Outcomes included indicators for depression screening, psychotherapy, pharmacotherapy, and combined psychotherapy-pharmacotherapy, separately for the first, second, and third trimesters, and 2 and 6 months postpartum. This study utilized a difference-in-differences approach that compared pre-post change in an outcome for the policy group to a counterfactual pre-post change from the comparison group.

**Results:**

Medicaid expansion led to a 3.64%-point increase in the rate of depression screening 6 months postpartum, 3.28%-point increase in the rate of psychotherapy 6 months postpartum, and 2.3 and 1%-point increases in the rates of pharmacotherapy and combined treatment in the first trimester, respectively. The relationships were driven by disproportionate gains among non-Hispanic whites and urban residents.

**Conclusions:**

Expanding Medicaid eligibility may improve depression screening and treatment among low-income women early in pregnancy and/or beyond the usual two-month postpartum period. However, it does not necessarily reduce racial/ethnic and regional gaps in depression screening and treatment.

## Introduction

Depression is highly prevalent, chronic, and costly (1–4). The 12-month prevalence of major depression was 8.7% for women in 2017, compared to 5.3% for men (5). As the most common complication of childbearing (1, 6), depression affects 8.5–11.5% of pregnant women (7), and 11–14% women are affected by postpartum depression (8–11). Low-income mothers of infants and young children are particularly vulnerable: over 10% of poor infants have a mother who is severely depressed and more than half have a mother with some depression (12). Also, new mothers with depression experience more severe, chronic symptoms if they remain untreated or undertreated (13).

Access to appropriate treatment of depression remains limited for low-income adults, especially among racial minority groups (14, 15). In 2008–2010, about half (49.4%) of uninsured low-income mothers of young children who had a major depressive episode did not receive any treatment for their depressive condition and 70.7% of them did not use prescription medication (16). Lack of health insurance coverage is arguably among the main reasons for the low rates of depression screening and treatment among low-income women (17).

As the largest funding source for perinatal and maternity care in the U.S., Medicaid offers low-income women a wide range of behavioral services, such as mental health counseling, psychopharmacology, crisis stabilization, and substance or alcohol abuse treatment (18). Despite state-level variations in the scope and type of services, Medicaid programs must provide comprehensive benefits for pregnancy-related services (e.g., prenatal care, counseling and support services, delivery, postpartum visits, substance use disorder services, and home visits) to pregnant women with incomes up to 133% of the federal poverty level (FPL), and cover them for 60 days postpartum (18). However, categorical eligibility requirements (such as the elderly, individuals with disabilities, children, and pregnant women) disrupt continuous health insurance coverage, providing many women with Medicaid benefits only during pregnancy and up to 60 days after delivery. Recent data show that about one-third of Medicaid women with a live birth in 2018 lost Medicaid eligibility within 6 months postpartum (19). In 2014, the Affordable Care Act (ACA) broadened Medicaid eligibility by allowing states to provide continuous full Medicaid benefits to individuals with modified adjusted gross income (MAGI) at or below 138% FPL, regardless of pregnancy status, through an optional Medicaid expansion, which extended coverage for many low-income mothers beyond pregnancy and the immediate 2-month postpartum period (18). As of June 2022, 39 states (including the District of Columbia) had extended Medicaid eligibility under the ACA (20). By 2016, 9.5 million low-income women who were not categorically eligible prior to the ACA obtained continuous Medicaid coverage through the Medicaid expansion (21). Further, under the ACA state Medicaid programs must cover preventive care recommended by the United States Preventive Services Task Force (USPSTF) for beneficiaries who become eligible under ACA Medicaid expansion. Specifically, the Alternative Benefit Plan (ABP) that must be provided under the Medicaid expansion option includes 10 Essential Health Benefits, such as depression screening and services for mental health and substance use disorders (18). For example, the USPSTF recommends screening for depression in the general adult population (including pregnant and postpartum women) (22) and counseling interventions for perinatal depression (23). The ACA Medicaid expansion, therefore, presents a unique opportunity to improve the screening and treatment of depression among low-income women in pregnancy and postpartum.

This study examined the extent to which the ACA Medicaid expansion in Oregon led to improved screening and treatment for depression among pregnant and postpartum women who had Medicaid-financed births. We posit that Medicaid expansion increased access to depression screening and treatment among Medicaid-eligible women, especially early in pregnancy and beyond 2 months from delivery, because it extended coverage for low-income mothers beyond pregnancy and the immediate 2-month postpartum period. We also explored potential heterogeneity in the effect of Medicaid expansion by race/ethnicity and rurality of residence location. Individuals from racial/ethnic minority groups and rural settings tend to have lower incomes. They are also more likely to receive Medicaid benefits, and financial barriers often interfere with their access to mental health treatment (24–27). Therefore, results may hold policy implications for reducing a gap in access to depression treatment for low-income racial/ethnic minority and rural populations.

## Methods

### Data sources and sample

We retrieved data from Oregon birth certificates and Medicaid Management Information System (MMIS). Birth certificate data included all Oregon live births between January 2011 and December 2016 (*N* = 266,600; 207,356 women). We restricted analysis to women who became pregnant on or after January 1, 2011 and gave birth on or before July 1, 2013 (to allow for six-month postpartum follow-up) for the pre-expansion period, and then conceived again on or after January 1, 2014 and gave birth on or before July 1, 2016 for the post-Medicaid expansion period. All women in the birth certificate data were deterministically linked to Medicaid enrollment and claims data from the MMIS. The enrollment data were used to identify sources of payment at delivery. The claims data provided diagnosis and procedure codes we used to construct outcome measures, shown below.

This study measured the effect of ACA Medicaid expansion on those targeted by the provision, women who otherwise might have received health insurance benefits through pregnancy-only Medicaid. Using the Medicaid enrollment data, we identified a policy group of 1,368 women (*n* = 2,831) who were covered by pregnancy-only Medicaid at childbirth in the pre-expansion period but had full-scope Medicaid coverage at childbirth in the post-expansion period. The comparison group included 2,229 women (*n* = 4,580) covered by full-scope Medicaid at childbirth in both pre- and post-expansion periods. So, the study sample included a total of 3,597 women (*n* = 7,411) in Oregon Medicaid who gave births financed by Medicaid in both the pre-expansion (2011–2013) and post-expansion (2014–2016) periods. The study protocol was approved by Institutional Review Boards at the Oregon Health Authority (IRB No. 13–25) and Oregon State University.

### Outcome measures

We utilized 20 binary outcome measures. We created depression screening indicators, separately for the first, second, and third trimesters of pregnancy and 2- and 6-month postpartum periods. Depression screening was identified by diagnoses of screening for depression, Current Procedural Terminology (CPT) codes, or Healthcare Common Procedure Coding System (HCPCS) codes in Medicaid medical claims data (details are available in Appendix Table 1).

Treatment of depression was categorized into psychotherapy only, pharmacotherapy (antidepressants) only, and combined treatment with psychotherapy and antidepressant medication. As with screening above, we constructed five binary indicators for each outcome category. We identified psychotherapy with CPT codes in combination with depression diagnoses. We identified pharmacotherapy with national drug code (NDC) and included all classes of antidepressant drugs: selective serotonin reuptake inhibitors (SSRI), selective norepinephrine reuptake inhibitors (SNRI), tricyclic antidepressants (TCA), monoamine oxidase inhibitors (MAOIs), and other depressants (mirtazapine and bupropion) (see Appendix Table 1 for details).

### Statistical analysis

We utilized a difference-in-differences (DID) approach that compared the pre-post difference in the rates of depression screening and treatment for the policy group to the counterfactual pre-post difference in that outcome for the comparison group (see Appendix A). The main independent variable was the interaction term of the policy-group and post-expansion period indicators; its coefficient captures an incremental change in the outcome attributable to Medicaid expansion. The post-expansion period indicator was included in the model to adjust for average pre-post trends for each outcome. We controlled for maternal characteristics including age at childbirth, race/ethnicity, education, marital status, rurality, body mass index (BMI) at childbirth, smoking during pregnancy, and history of multiple births (e.g., twins and triplets). Race/ethnicity categories included non-Hispanic white (reference), black, American Indian/Alaska Native, Asian, Native Hawaiian or Pacific Islander, and Hispanic. Rurality was specified as urban areas (reference category), large rural areas, or small/isolated rural areas, which we defined using ZIP codes of residence and Rural Urban Commuting Area (RUCA) criteria (28). We included year-month linear time trend and month dummies to control for secular time trends and seasonality, respectively. Also, individual fixed-effects controlled for all time-invariant unobserved heterogeneity (e.g., unobserved health conditions that might affect both depression and Medicaid eligibility) so that our estimates have a stronger robust property arising from minimizing the influence of unobserved characteristics unique to each individual.

To explore racial/ethnic heterogeneity, we augmented the reference model with additional triple interaction terms obtained by multiplying the main interaction term by each race/ethnicity category. To explore the effects of Medicaid expansion by the rurality of residence we included additional triple interaction terms for rurality, separately for large and small/isolated rural areas. We computed incremental effects separately for each racial/ethnic group and rurality category as a linear combination of coefficients (see Appendix A for details).

Our DID estimates may capture unbiased effects of the Medicaid expansion because of the assumption of parallel trends in the outcomes in the pre-expansion period. Examination of aggregate monthly trends indicates that the policy and control groups exhibited closely-overlapped pre-expansion trends for all the outcomes (see Appendix Figure 1). Results from falsification regression analyses also confirmed that the parallel trend assumption was satisfied (see Appendix B).

We estimated linear probability models, so our estimates can be directly interpreted as average incremental changes in the outcomes over the post-expansion period. Over 98% of predicted probabilities ranged within the unit interval. Standard errors were adjusted for intraclass correlation at the person level.

## Results

Compared to women in the comparison group (those covered by full-scope Medicaid at childbirth in both pre- and post-expansion periods), women in the policy group (those covered by pregnancy-only Medicaid at childbirth before expansion but full-scope Medicaid at childbirth after expansion) were more likely to be non-Hispanic white, educated, married, a rural resident, and a smoker (Table 1). Mean age and mean BMI were similar between the groups. The proportion of multiple-birth history was also comparable and had similar pre-post increases for both groups.

**Table 1:** Characteristics of the study sample (*n* = 7,411).

**Characteristic**	**Policy group**		**Comparison group**	
	**Pre**	**Post**	**Pre**	**Post**
	**%**	**%**	**%**	**%**
**Main explanatory variables**				
Policy group^a^	100	100	0	0
Post period^b^	0	100	0	100
Policy groupXpost period^c^	0	100	0	0
**Covariates**
Mother's age at delivery [mean (SD)]	24.2 (4.98)	27.0 (4.97)	25.0 (5.48)	27.8 (5.41)
**Race/ethnicity**				
White (reference)	67.1	67.5	31.8	32.3
Black	4.09	4.03	5.57	5.52
AIAN^d^	6.63	6.23	4.52	4.70
Asian	1.90	1.91	1.97	1.91
NHPI^e^	1.20	1.27	2.46	2.52
Hispanic	19.1	19.0	53.7	53.1
**Education level**				
< High school (reference)	23.3	18.7	49.3	45.2
High school graduates	65.2	67.3	45.7	49.2
College and higher	11.5	14.0	5.07	5.63
Mother married at delivery	37.7	44.3	35.4	43.3
**Rurality of residence location**				
Urban resident (reference)	85.2	79.2	87.1	81.8
Large rural resident	10.3	15.6	8.81	13.7
Small/remote rural resident	4.49	5.23	4.13	4.45
BMI at delivery [mean (SD)]	32.1 (6.53)	32.7 (6.83)	32.2 (6.25)	32.9 (6.56)
Smoked during pregnancy	21.5	21.3	16.7	19.8
History of multiple births	0.53	1.77	0.50	1.61
*n*	1,418	1,413	2,280	2,300

^a^Policy group consists of women covered by pregnancy-only Medicaid before Medicaid expansion and full-scope Medicaid after Medicaid expansion. Reference group consists of women covered by full-scope Medicaid both before and after Medicaid expansion.

^b^Post-Medicaid expansion period indicator for 2014–2016.

^c^Policy groupXpost period = Interaction of the policy group and post-expansion period indicators.

^d^AIAN, American Indian/Alaskan Native.

^e^NHPI, Native Hawaiian/Pacific Islander.

Women in the policy and comparison groups had similar rates of depression screening and treatment at baseline (i.e., before Medicaid expansion), and both groups experienced increases in the rates following the Medicaid expansion (Table 2). Unadjusted difference-in-differences estimates were almost always positive and the largest in the first trimester for pharmacotherapy and in the 6-month postpartum period for screening, psychotherapy, and combined treatment. This finding indicates overall larger increases in the crude rates of depression screening and treatment for the policy group following the Medicaid expansion. Appendix Figure 1 illustrates the patterns using month-to-month aggregate time-series for each outcome.

**Table 2:** Outcome measures.

**Characteristic**	**Policy group**		**Comparison group**		**Crude DID estimates^a^**
	**Pre**	**Post**	**Pre**	**Post**	
	**%**	**%**	**%**	**%**	
**Depression screening**					
First trimester	11.8	13.9	12.6	14.1	0.6
Second trimester	12.1	12.9	12.3	14.7	−1.5
Third trimester	22.1	20.0	19.9	21.9	−4.1
Two–month postpartum	7.62	12.4	6.93	9.74	2.0
Six–month postpartum	14.7	23.1	13.8	18.7	3.5
**Treatment for depression**					
**Psychotherapy only**					
First trimester	10.4	9.70	10.5	11.4	−1.7
Second trimester	10.6	11.0	10.9	12.5	−1.1
Third trimester	20.3	17.4	18.1	19.7	−4.5
Two–month postpartum	4.72	7.22	4.61	6.39	0.7
Six–month postpartum	7.83	12.5	8.07	10.5	2.3
**Pharmacotherapy only**					
First trimester	1.97	4.60	2.37	2.70	2.3
Second trimester	2.75	4.53	1.97	2.52	1.2
Third trimester	2.82	4.53	2.28	2.87	1.1
Two–month postpartum	7.48	9.77	5.18	5.96	1.5
Six–month postpartum	10.2	13.2	7.54	9.22	1.3
**Combined treatment**					
First trimester	0.56	1.77	1.01	1.26	1.0
Second trimester	0.85	1.42	0.66	1.13	0.1
Third trimester	1.34	2.26	1.05	1.43	0.5
Two–month postpartum	1.41	2.62	1.05	1.00	1.3
Six–month postpartum	3.03	6.37	2.94	4.17	2.1

^a^Reported are unadjusted difference-in-differences estimates in the effect of Medicaid expansion.

Reported in Table 3 are adjusted difference-in-difference estimates which measure incremental effects of Medicaid expansion (see Appendix Tables 2–5 for full results). Medicaid expansion was not significantly associated with a change in depression screening in pregnancy and 2 months postpartum. However, it was associated with a 3.64%-point increase in the rate of depression screening 6 months postpartum (*p* = 0.047). This change represents an increase by one quarter from the pre-expansion average of 14.7% for the policy group.

**Table 3:** Effects of Medicaid expansion on depression screening and treatment among women enrolled in Oregon Medicaid (incremental changes in percentage points)^a^.

**Perinatal period**	**Screening**	**Treatment**		
		**Psychotherapy**	**Pharmacotherapy**	**Combined treatment**
First trimester	−1.20 (1.57)	−2.50 (1.49)	2.30** (0.83)	0.98* (5.01)
Second trimester	−1.47 (1.64)	−0.78 (1.55)	1.34 (0.80)	0.34 (0.49)
Third trimester	−3.10 (1.96)	−3.70 (1.90)	0.82 (0.77)	0.84 (0.59)
Two–month postpartum	2.19 (1.48)	1.29 (1.20)	0.75 (1.14)	1.12 (0.60)
Six–month postpartum	3.64* (1.83)	3.28** (1.49)	−0.14 (1.43)	1.83 (0.98)

^a^Reported are difference-in-differences estimates of incremental effects of Medicaid expansion measured in percentage-point changes. All models are fully specified and the full set of regression results is reported in Appendix Tables 2–5. Standard errors in parentheses are adjusted for intraclass correlation.

*p < 0.05.

**p < 0.01.

Medicaid expansion was not associated with changes in psychotherapy rates in pregnancy and 2 months postpartum, but was significantly associated with a 3.28**%**-point increase in the rate of psychotherapy 6 months postpartum (*p* < 0.01), a 42% increase from the pre-expansion average of 7.83% for the policy group. The rates of pharmacotherapy and combined treatment significantly increased in the first trimester by 2.3**%** points (*p* < 0.01) and 1**%** point (*p* = 0.049), respectively, following Medicaid expansion.

Table 4 presents the effect of ACA Medicaid expansion by race/ethnicity. We report incremental effects for each racial/ethnic subgroup, computed as linear combination of coefficients (see Appendix Tables 6–9 for regression coefficients). Significant increases in screening and treatment of depression following Medicaid expansion, when there were such increases, were found mostly for non-Hispanic white women: Medicaid expansion was associated with an increase in the rate of screening in the first trimester (2.34**%**-point increase, *p* = 0.039); psychotherapy 6 months postpartum (3.54**%**-point increase, *p* = 0.036); pharmacotherapy in the first trimester (3.05**%**-point increase, *p* < 0.001) and the second trimester (1.91**%**-point increase, *p* = 0.035); and combined treatment in the first trimester (1.11**%**-point increase, *p* = 0.049), 2 months postpartum (1.42**%**-point increase, *p* = 0.032), and 6 months postpartum (2.34**%**-point increase, *p* = 0.039). Significant positive effects of Medicaid expansion were also found for American Indians/Alaska Natives in a few instances such as the rate of screening 6 months postpartum (13.8**%**-point increase, *p* = 0.012); pharmacotherapy 6 months postpartum (8.56**%**-point increase, *p* = 0.046); and combined treatment in the first trimester (4.04**%**-point increase, *p* < 0.01).

**Table 4:** Effects of Medicaid expansion on depression screening and psychotherapy use among women enrolled Oregon Medicaid by race/ethnicity (incremental changes in percentage points)^a^.

**Perinatal period and race/ethnicity**	**Screening**	**Treatment**		
		**Psychotherapy**	**Pharmacotherapy**	**Combined treatment**
**First trimester**
Non-Hispanic White	2.34* (1.13)	−2.83 (1.69)	3.05*** (0.94)	1.11* (0.58)
Black	3.99 (6.36)	0.45 (5.85)	−0.44 (3.27)	−0.12 (2.04)
AIAN^b^	−0.92 (4.85)	−2.05(4.47)	0.89 (2.49)	4.04** (1.56)
Asian	−7.07 (8.63)	−8.76 (7.94)	4.59 (4.43)	−0.39 (2.77)
NHPI^c^	−2.01 (11.1)	−1.44 (2.80)	−0.56 (5.69)	−0.46 (3.56)
Hispanic	−1.29 (3.04)	−2.14 (2.68)	0.65 (2.56)	0.07 (0.98)
**Second trimester**
Non-Hispanic White	0.31 (1.83)	−0.78 (1.76)	1.91* (0.91)	0.33 (0.54)
Black	−5.32 (6.52)	−4.46 (6.11)	−0.26 (3.15)	−0.24 (1.88)
AIAN^b^	−4.55 (4.98)	−2.69 (4.66)	3.00 (2.40)	−0.12 (1.43)
Asian	−2.20 (8.84)	−1.10 (8.19)	2.23 (4.28)	−0.33 (2.55)
NHPI^c^	−9.28 (11.4)	−8.97 (10.6)	−0.36 (5.49)	−0.54 (3.28)
Hispanic	0.16 (3.12)	1.28 (2.92)	−0.94 (1.51)	0.10 (0.90)
**Third trimester**
Non-Hispanic White	−1.15 (1.88)	−3.52 (2.16)	1.57 (0.87)	1.20 (0.66)
Black	−1.99 (7.77)	−5.45 (7.15)	−4.51 (3.04)	1.46 (2.30)
AIAN^b^	−9.41 (5.93)	−7.78 (5.73)	2.99 (2.32)	0.86 (1.76)
Asian	−14.2 (10.5)	−13.4 (10.2)	2.23 (4.28)	−0.57 (3.13)
NHPI^c^	−4.35 (13.5)	−3.72 (13.1)	−0.46 (4.12)	−0.13 (4.02)
Hispanic	2.41 (3.72)	−1.71 (3.59)	−0.03 (1.45)	−0.34 (1.10)
**Two–month postpartum**
Non-Hispanic White	−2.42 (2.24)	1.38 (1.36)	−0.28 (1.30)	1.42* (0.66)
Black	0.18 (5.66)	−0.26 (4.73)	2.76 (4.49)	−0.09 (2.31)
AIAN^b^	4.52 (4.32)	1.82 (3.62)	11.4 (3.43)	0.23 (1.76)
Asian	4.90 (7.68)	2.82 (6.42)	2.93 (6.10)	−0.26 (3.13)
NHPI^c^	−2.34 (9.87)	5.49 (8.25)	−2.34 (7.83)	−0.09 (4.02)
Hispanic	2.16 (2.70)	0.64 (2.26)	0.23 (2.15)	0.82 (1.10)
**Six–month postpartum**
Non-Hispanic White	2.06 (1.63)	3.54* (1.70)	−1.32 (1.62)	2.34* (1.13)
Black	−2.65 (7.15)	0.40 (5.87)	1.69 (5.63)	1.42 (3.93)
AIAN^b^	13.8* (5.46)	6.82 (4.48)	8.56* (4.29)	0.34 (3.30)
Asian	3.78 (9.71)	1.41 (7.97)	6.97 (7.63)	1.41 (5.33)
NHPI^c^	−5.03 (12.4)	11.6 (10.2)	8.10 (9.80)	−1.52 (6.84)
Hispanic	1.93 (3.42)	1.26 (2.81)	−0.79 (2.69)	0.89 (1.88)

^a^Reported are difference-in-differences estimates of incremental effects of Medicaid expansion measured in percentage-point changes. They came from the augmented model that included additional triple interaction terms of the policy group indicator, post-ACA period indicator, and race/ethnicity categories. See Appendix Tables 6–9 for full coefficients. All covariates were included and standard errors are adjusted for intraclass correlation. The incremental effect was calculated for each race/ethnicity subgroup as the linear combination of coefficients for the reference group (whites) plus the interaction term coefficient for the race/ethnicity category.

^b^AIAN, American Indian/Alaskan Native.

^c^NHPI, Native Hawaiian/Pacific Islander.

*p < 0.05.

**p < 0.01.

***p < 0.001.

The effects of Medicaid expansion by rurality are reported in Table 5 (see Appendix Tables 10–13 for regression coefficients). Statistically significant increases in the rates of depression screening and treatment following Medicaid expansion were found among urban residents, when there were such increases as the rates of screening 2 months postpartum (2.99**%**-point increase, *p* = 0.050) and 6 months postpartum (4.54**%**-point increase, *p* = 0.018); psychotherapy 6 months postpartum (3.67**%**-point increase, *p* = 0.020); pharmacotherapy in the first trimester (2.16**%**-point increase, *p* = 0.014) and the second trimester (1.70**%**-point increase, *p* < 0.01); and combined treatment 2 months postpartum (1.64**%**-point increase, *p* < 0.01) and 6 months postpartum (2.48**%**-point increase, *p* = 0.019). For women living in large rural regions, only the rate of pharmacotherapy increased by 5.71**%** points following Medicaid expansion. Interestingly, Medicaid expansion was associated with decreases in the rates of screening (11.6**%**-point decrease, *p* = 0.021) and psychotherapy (8.51**%**-point decrease, *p* = 0.044) 2 months postpartum among women in small rural areas. Otherwise, no statistically detectable effect of Medicaid expansion was discovered by the level of rurality.

**Table 5:** Effects of Medicaid expansion on depression screening and psychotherapy use among women enrolled Oregon Medicaid by the rurality of residence (incremental changes in percentage points)^a^.

**Perinatal period and rurality**	**Screening**	**Treatment**		
		**Psychotherapy**	**Pharmacotherapy**	**Combined treatment**
**First trimester**
Urban	−0.54 (1.71)	−2.62 (1.58)	2.16* (0.88)	0.92 (0.55)
Large rural	4.86 (3.93)	−0.25 (3.61)	5.71** (2.02)	1.11 (1.26)
Small rural	−4.03 (5.67)	−4.21 (5.00)	−2.61 (2.91)	1.69 (1.82)
**Second trimester**
Urban	−1.68 (1.76)	−0.55 (1.64)	1.70* (0.85)	0.09 (0.51)
Large rural	−0.92 (4.03)	−1.98 (3.78)	−2.20 (1.95)	2.17 (1.17)
Small rural	0.51 (5.82)	−1.93 (5.45)	3.21 (2.81)	0.27 (1.69)
**Third trimester**
Urban	−2.46 (2.09)	−3.39 (2.02)	1.07 (0.82)	0.89 (0.62)
Large rural	−4.92 (4.80)	−4.99 (4.64)	−0.73 (1.88)	1.97 (1.42)
Small rural	−9.12 (6.92)	−5.93 (6.70)	0.22 (2.72)	−2.22 (2.06)
**Two–month postpartum**
Urban	2.99* (1.52)	1.72 (1.27)	0.88 (1.21)	1.64** (0.62)
Large rural	2.82 (3.50)	2.78 (2.92)	−3.05 (2.78)	−0.97 (1.42)
Small rural	−11.6* (5.05)	−8.51* (4.22)	6.67 (4.01)	−2.59 (2.05)
**Six–month postpartum**
Urban	4.54* (1.93)	3.67* (1.58)	−0.46 (1.52)	2.48* (1.06)
Large rural	3.32 (4.43)	4.76 (3.63)	−1.18 (3.48)	−0.18 (2.42)
Small rural	−9.83 (6.38)	−5.94 (5.23)	6.95 (5.02)	−4.07 (3.50)

^a^Reported are difference-in-differences estimates of incremental effects of Medicaid expansion measured in percentage-point changes. They came from the augmented model that included additional triple interaction terms of the policy group indicator, post-ACA period indicator, and rurality categories. See Appendix Tables 10–13 for full coefficients. All covariates were included and standard errors are adjusted for intraclass correlation. The incremental effects presented here for each category of rurality was computed as the linear combination of coefficients for the reference group (urban) plus the interaction term coefficient for the rurality category.

*p < 0.05.

**p < 0.01.

## Discussion

This study tested whether ACA Medicaid expansion led to a change in screening and treatment of depression among pregnant and postpartum women who gave births financed by Oregon Medicaid. Our analyses provide evidence that Medicaid expansion in Oregon led to non-trivial increases in the rates of screening and treatment for depression in the first trimester and/or 6 months postpartum among women who had Medicaid-financed deliveries. We interpret the findings as a beneficial effect of extending insurance coverage for low-income women under Medicaid expansion early in their pregnancies and beyond the usual 2-month postpartum period. This finding is encouraging because timely access to care, especially at the early stages of pregnancy, is among the important determinants of maternal and infant health (29), and perinatal depression can potentially lead to a variety of adverse outcomes for both mother and infant (30–32).

Our findings add to the emerging research in this area that documents beneficial impacts of expanding Medicaid programs on access to mental health services among low-income individuals. Fry and Sommers (33) reported a 23**%**-point reduction in the rate of uninsured low-income adults with depression as well as improvement in access to health care and medication in Arkansas and Kentucky following Medicaid expansion in those states. Baicker et al. (17) found a 50% reduction in undiagnosed depression and 60% reduction in untreated depression among previously uninsured adults in Oregon following the state's Medicaid expansion through Oregon's Medicaid lottery in 2008.

Although it goes beyond the scope of the current analysis, we speculate that ACA Medicaid expansion might ultimately lead to a decline in maternal mortality and morbidity among low-income women given that depression has been verified as a major risk factor for perinatal suicidality, with risk being elevated by the severity of symptoms (34, 35). The prevalence of suicidality among women with minor depression was 34.1% during pregnancy and 30.6% during the postpartum period (36). Postpartum depression, which influences ~10–15% of women who deliver worldwide (37), is a strong predictor of postpartum suicidality (38). In addition, the beneficial effect of Medicaid expansion might also extend to infants and young children of low-income mothers with depression. When it remains untreated, maternal depression can lead to adverse infant outcomes (39), and an elevated risk of physical, mental, cognitive, socioemotional, and behavioral problems among children over the longer term (40, 41). We also infer that Medicaid expansion may ultimately reduce a gender gap in depression prevalence (1, 5) and income-based disparities in treatment (42). Future work may assess the validity of this conjecture.

The literature points to significant racial/ethnic disparities in treatment of psychiatric disorders including depression (42–44), despite lack of evidence on racial/ethnic differences in the prevalence of depression (45). Further, recent studies have reported widening or sustained gaps in mental health treatment (including detection and treatment of depression) between non-Hispanic whites and racial/ethnic minority populations (42, 44, 46, 47). Rurality of residence location also deserves attention because disparities in access to depression treatment between urban and rural residents have been reported (43). In one study, rates of mental health treatment decreased gradually as the rurality of residence location increased from large metropolitan areas to isolated rural settings (48). Our findings suggest that expanding Medicaid does not necessarily contribute to a reduction of existing racial/ethnic and regional gaps in screening and treatment of depression in pregnancy and postpartum among Medicaid-eligible low-income women. Further, eliminating such disparities requires more than simply expanding population health insurance coverage. We also need to confront other system and social determinants of mental health care disparities, such as the supply of racial/ethnic minority and rural mental health providers and training a culturally-competent mental health workforce to address unique circumstances of minority patients such as cultural values (44).

Data come from Oregon's on-going efforts to transform its Medicaid program offer a potential promise. Since 2012 Coordinated Care Organizations (CCOs) have served Oregon Medicaid beneficiaries across the state, prioritizing the elimination of health disparities through a multifaceted approach that included community health workers, Regional Health Equity Coalitions, and integration of physical and behavioral health care. Early data show that CCO implementation was associated with reductions in racial differences in access to care (49). In January 2020, the state launched the second round of the system transformation through CCO 2.0 (50), which further emphasizes improvement in the behavioral health system and focuses on social determinants of health and health equity. We believe that such system transformation, along with the expansion of behavioral health benefits coverage, is necessary to address perinatal mental health disparities because simply expanding population behavioral health insurance coverage does not guarantee existing disparities in access to depression screening and treatment for low-income pregnant and postpartum women.

Limitations are noteworthy. First, we examined only women in the pregnancy and 6-month postpartum periods, and therefore our findings should be generalized to the other low-income women with caution. Second, we restricted our analysis to women who gave births financed by Medicaid in both pre- and post-expansion periods to elevate the internal validity of findings. This approach, however, might weaken the external validity, and therefore our results should be generalized to the entire Medicaid women with caution. Nonetheless, the entire population of Oregon women who gave live births during the study period were quite similar to those included in our study in terms of many observable characteristics (see Appendix Table 14). Third, because our findings reflect one state's experience of Medicaid expansion, they may not be generalizable to other states that differ in terms of demographics, population health, political climate, or how Medicaid program structure. Fourth, we did not have information on depression screening and treatment for women who were uninsured or privately-insured. Therefore, our findings should be viewed from the perspective of the Medicaid program, and our estimates might be greater in magnitude than those from the societal perspective. However, we do not expect that our estimates are significantly overstated because uninsured women in general do not receive adequate screening and treatment for depression, and privately-insured women in general are not affected by Medicaid expansion.

## Conclusions

This study provides evidence of the value of expanding a Medicaid program for low-income women of reproductive age, showing that expanding Medicaid eligibility could offer an opportunity to improve depression screening and treatment among low-income women early in pregnancy and/or beyond postpartum. Our findings also underscore the need for further addressing racial/ethnic and regional gaps in depression screening and treatment.

## Data availability statement

The datasets presented in this article are not readily available because the current Data Use Agreement between Oregon State University (OSU) and Oregon Health Authority (OHA) does not allow public release of any data files including original data from OHA and analytic data files constructed by OSU. Requests to access the datasets should be directed to marie.harvey@oregonstate.edu.

## Ethics statement

The studies involving human participants were reviewed and approved by Institutional Review Boards at the Oregon Health Authority and Oregon State University Institutional Review Board. Written informed consent for participation was not required for this study in accordance with the national legislation and the institutional requirements.

## Author contributions

JY conceived and designed the study, performed statistical analysis, and drafted the manuscript. SH, JL, and JY acquired data and revised the manuscript for important intellectual content. All authors have read and approved the submitted version.

## Funding

This work was supported through the Centers for Disease Control and Prevention's Cooperative Agreement 1U01DP004783-01 to the College of Public Health and Human Sciences at Oregon State University.

## Conflict of interest

The authors declare that the research was conducted in the absence of any commercial or financial relationships that could be construed as a potential conflict of interest.

## Publisher's note

All claims expressed in this article are solely those of the authors and do not necessarily represent those of their affiliated organizations, or those of the publisher, the editors and the reviewers. Any product that may be evaluated in this article, or claim that may be made by its manufacturer, is not guaranteed or endorsed by the publisher.

## Author disclaimer

The contents, views or opinions expressed in this publication are those of the authors and do not necessarily reflect official policy or position of Uniformed Services University of the Health Sciences, the Department of Defense (DoD), or Departments of the Army, Navy, or Air Force.
