# Supplementary material for: Improved depression screening and treatment among low-income pregnant and postpartum women following Medicaid expansion in the U.S
Source: Front Health Serv. 2022 Aug 17;2:942476. doi: 10.3389/frhs.2022.942476 (PMC10012772; doi:10.3389/frhs.2022.942476)
Supplement: Supplementary file 1 [file Data_Sheet_1.docx]

***Supplementary Material***

**Appendix Table 1. Definitions of depression screening and treatment**

| **Outcome** | **Definition** | **Diagnosis** | **CPT** | **HCPCS** |
| --- | --- | --- | --- | --- |
| Depression screening | = 1 if received screening for depression based on diagnoses, Current Procedural Terminology (CPT), and Healthcare Common Procedure Coding System (HCPCS) codes, and 0 if not. | ICD-9-CM V79.0; ICD-10-CM Z13.89 (screening for depression) | 3725F (screening for depression performed).  The following CPT codes [before / after 2013 revision] used in combination with depression diagnoses^a^:  90801, 90802 / 90791, 90792 (Psychotherapy diagnostic interview);  99201-99205 (office/outpatient visits for the evaluation and management of a new patient);  99212-99215 (office/outpatient visits for the evaluation and management of an established patient);  96150, 96151 (health and behavior assessment and intervention);  99385-99387 (initial comprehensive preventive medicine evaluation and management); and  99395-99397 (periodic comprehensive preventive medicine re-evaluation and management). | HCPCS codes for G0444 (15-minute annual depression screening); G8431-G8433, G8510, G8511, G8940 (depression screening results); and  G0438, G0439 (annual wellness visits) in combination with depression diagnoses^a^. |
| Psychotherapy | = 1 if received psychotherapy treatment for depression, based on CPT codes, and did not receive pharmacotherapy, and 0 if not. |  | CPT codes [before 2013 / after 2013 revision] in combination with depression diagnoses^a^ as following:  90804, 90805, 90816, 90817 / 90832, 90833 (individual psychotherapy 20–30 minutes);  90806, 90818, 90807, 90819 / 90834, 90836 (individual psychotherapy 45–50 minutes);  90808, 90821, 90809, 90822 / 90837, 90838, 90847 (individual psychotherapy 75–80 minutes);  90810, 90811, 90823, 90824 / 90832, 90833 (interactive individual psychotherapy 20–30 minutes);  90812, 90813, 90826, 90827 / 90834, 90836 (interactive individual psychotherapy 45–50 minutes);  90814, 90815, 90828, 90829 / 90836, 90837, 90838 (interactive individual psychotherapy 75–80 minutes);  90846, 90847, 90849 / 90846, 90847, 90849 (family psychotherapy);  90853 / 90853 (group psychotherapy);  90857 / 90853 (interactive group psychotherapy); and  - / 90839, 90840 (psychotherapy for crisis). |  |
| Pharmacotherapy | = 1 if received any antidepressant but did not receive psychotherapy, and 0 if otherwise; based on national drug codes in pharmacy claims including antidepressant drugs: selective serotonin reuptake inhibitors (SSRI), selective norepinephrine reuptake inhibitors (SNRI), tricyclic antidepressants (TCA), monoamine oxidase inhibitors (MAOIs), and other depressants (mirtazapine and bupropion). |  |  |  |
| Combined treatment | = 1 if received both psychotherapy and pharmacotherapy, and 0 if not. |  |  |  |

^a^Depression was identified using the following diagnoses codes for major depression, minor depression, and postpartum depression: ICD-9-CM codes of 296.2, 296.21-296.26, 296.3, or 296.31-296.36, 296.82, 298, 300.4, 309.1, 309.28, and 311; and ICD-10-CM codes of F320-F325, F329, F330-F333, F339, F341, F342, and F4321.

**Appendix A. Difference-in-differences model specifications**

The main regression model takes the following empirical form:

(A1) $Pr(u_{it}>0)=\beta_{1}e_{i}\times p_{t}+\beta_{2}p_{t}+{x_{it}^{'}\beta}_{3}+\beta_{4}t_{t}+m_{t}^{'}\beta_{5}+\alpha_{i}+\varepsilon_{i}$

where *i and t subscripts* denote individual and year-month, respectively, and the $\beta$’s are the coefficients to be estimated. The dependent variable ($u$) is each of the binary outcome measures (depression screening, psychotherapy, pharmacotherapy, and combined treatment). We estimated the model separately for each of the five peripartum periods. The interaction term ($e\times p$) of the policy group ($e$) and post-expansion period ($p$) indicators is of primary interest as a significant and positive coefficient ($\hat{\beta}_{1}$) of the interaction term may indicate an increase in the probability of the outcome. We controlled in a vector $x'$ for time-varying maternal characteristics such as age, education, marital status, rurality, body mass index at the time of giving birth, smoking during pregnancy, partity, and plurality. We also specified a year-month time trend ($t$), a vector of month dummies ($m'$), and unobserved individual heterogeneity term ($\alpha$).

Racial/ethnic heterogeneity

To explore racial/ethnic heterogeneity in the effect of Medicaid expansion on each outcome, we augmented the main regression model by including a set of triple interaction terms ($e\times p\times r$) of the interaction term ($e\times p$) and each race/ethnicity category ($r^{j}$). The main regression model takes the following empirical form:

(A2) ${Pr(u}_{it}>0)=\beta_{1}e_{i}\times p_{t}+\sum_{j=1}^{J} \beta^{j}e_{i}\times p_{t}\times r_{r}^{j}+\beta_{2}p_{t}+{x_{it}^{'}\beta}_{3}+\beta_{4}t_{t}+m_{t}^{'}\beta_{5}+\alpha_{i}+\varepsilon_{i}$

The coefficient $\beta_{1}$ on the interaction term of the policy group and post-expansion period indicators ($e\times p$) now captures the effect of Medicaid expansion for non–Hispanic white women (the reference race/ethnicity category) and the coefficient on each triple interaction term $\beta^{j}$ measures a difference in the effect of Medicaid expansion between women in the $j$^th^ race/ethnicity group and non–Hispanic white women.

Rural/urban heterogeneity

We similarly augmented the main regression model by including a set of triple interaction terms ($e\times p\times ru$) of the interaction term ($e\times p$) and each rurality of residence category (${ru}^{j}$). The main regression model takes the following empirical form:

(A3) ${Pr(u}_{it}>0)=\beta_{1}e_{i}\times p_{t}+\sum_{j=1}^{J} \beta^{j}e_{i}\times p_{t}\times{ru}_{r}^{j}+\beta_{2}p_{t}+{x_{it}^{'}\beta}_{3}+\beta_{4}t_{t}+m_{t}^{'}\beta_{5}+\alpha_{i}+\varepsilon_{i}$

The coefficient on the interaction term ($e\times p$) now captures the effect of Medicaid expansion for women living in urban regions (the reference race category) and the coefficient on each triple interaction term measures a difference in the effect of Medicaid expansion between women in the $j$^th^ rural regions and urban women.

**Appendix Figure 1. Trends in the rates of depression screening and treatment, 2011-2016**

Panel A: Depression screening

Pre expansion

Post expansion

Pre expansion

Post expansion

Pre expansion

Post expansion

Post expansion

Pre expansion

Pre expansion

Post expansion

Panel B: Psychotherapy only

Post expansion

Pre expansion

Post expansion

Pre expansion

Post expansion

Pre expansion

Post expansion

Pre expansion

Post expansion

Pre expansion

Panel C: Pharmacotherapy only

Pre expansion

Post expansion

Pre expansion

Post expansion

Pre expansion

Post expansion

Post expansion

Pre expansion

Post expansion

Pre expansion

Panel D: Combined treatment (psychotherapy + pharmacotherapy)

Post expansion

Pre expansion

Post expansion

Pre expansion

Pre expansion

Post expansion

Post expansion

Pre expansion

Pre expansion

Post expansion

**Appendix B. Falsification analyses for parallel trend assumption**

The parallel trend assumption (or common trend assumption) is the fundamental assumption underlying the difference-in-differences analysis. It refers to the same trend in an outcome over time in the pre-policy time period (i.e., before ACA Medicaid expansion) between the policy and comparison groups. We performed two different regression-based falsification analyses to test whether the parallel trend assumption is satisfied in our data.

First, we examined the effect of a placebo-policy only using data for the pre-expansion period (years 2011-2013). The placebo-policy ($pp$) takes the value of 1 for July 2012 or later and 0 for January 2011-June 2012. We alternatively defined the placebo-policy such as January 2012 or later. Because our findings always remained robust, we report results from the main model below.

We estimated the following empirical model:

(B1) ${Pr(u}_{it}>0)=\beta_{1}e_{i}\times{pp}_{t}+\beta_{2}{pp}_{t}+{x_{it}^{'}\beta}_{3}+\beta_{4}t_{t}+m_{t}^{'}\beta_{5}+\alpha_{i}$

where *i and t subscripts* denote beneficiary and month, respectively, and the $\beta$’s are the coefficients to be estimated. The dependent variable ($u$) is one of the outcome measures. The interaction term ($e\times pp$) of the policy group ($e$) and placebo-expansion period indicators is of primary interest. Its insignificant coefficient ($\hat{\beta}_{1}$) would indicate that the parallel trend assumption before ACA Medicaid expansion between the policy and comparison groups are satisfied. As in the main empirical model, we specified a vector of the same covariates, a vector of year dummies, a vector of month dummies, and unobserved individual heterogeneity term.

Second, we estimated the following model again only on the pre-expansion subsample, replacing the main interaction term and the post-expansion period indicator with a linear time trend ($time$) and interaction term of the linear time trend and the policy group indicator ($e\times time$). An insignificant coefficient on the interaction term ($\hat{\beta}_{1}$) would indicate that the parallel trend assumption before ACA Medicaid expansion between the policy and comparison groups is satisfied.

(B2) ${Pr(u}_{it}>0)=\beta_{1}e_{i}\times{time}_{t}+\beta_{2}{time}_{t}+{x_{it}^{'}\beta}_{3}+\beta_{4}t_{t}+m_{t}^{'}\beta_{5}+\alpha_{i}$

The following table reports the main coefficient ($\hat{\beta}_{1}$)and its standard error from the falsification analyses. As shown, none of the main independent variables (placebo-policy and interaction of linear time trend and policy group indicator) is statistically significant, suggesting the parallel assumption is always satisfied in our data.

| Falsification analysis | Screening | Treatment | | |
| --- | --- | --- | --- | --- |
|  |  | Psycho-therapy | Pharmaco-therapy | Combined treatment |
| 1. Examine the effect of a placebo–policy on the pre–expansion subsample | | | | |
| First trimester | 0.118 | 0.07 | 0.03 | 0.01 |
|  | (0.10) | (0.10) | (0.02) | (0.01) |
| Second trimester | –0.08 | –0.05 | 0.001 | 0.004 |
|  | (0.09) | (0.07) | (0.01) | (0.01) |
| Third trimester | 0.07 | –0.01 | 0.01 | –0.004 |
|  | (0.11) | (0.11) | (0.01) | (0.01) |
| Two–month postpartum | –0.06 | –0.05 | 0.03 | 0.005 |
|  | (0.05) | (0.04) | (0.02) | (0.01) |
| Six–month postpartum | 0.06 | 0.14 | –0.01 | 0.01 |
|  | (0.10) | (0.10) | (0.02) | (0.02) |
| 1. Examine group–specific linear time trends on the pre–expansion subsample | | | | |
| First trimester | 0.012 | 0.005 | 0.001 | 0.000 |
|  | (0.01) | (0.01) | (0.00) | (0.00) |
| Second trimester | –0.01 | –0.003 | 0.00 | –0.002 |
|  | (0.01) | (0.01) | (0.00) | (0.00) |
| Third trimester | 0.004 | –0.001 | 0.00 | 0.002 |
|  | (0.01) | (0.01) | (0.00) | (0.00) |
| Two–month postpartum | –0.01 | –0.004 | 0.005 | 0.000 |
|  | (0.00) | (0.00) | (0.00) | (0.00) |
| Six–month postpartum | 0.001 | 0.01 | –0.001 | –0.002 |
|  | (0.01) | (0.01) | (0.00) | (0.00) |

**Appendix Table 2. Effect of Medicaid expansion on depression screening** **among perinatal women in Oregon Medicaid: coefficients^a^**

|  | Perinatal Period: | | | | |
| --- | --- | --- | --- | --- | --- |
|  | First trimester | Second trimester | Third trimester | Two months postpartum | Six months postpartum |
| Policy groupXpost period | –0.0012 | –0.0147 | –0.0310 | 0.0219 | 0.0364^*^ |
|  | (0.0157) | (0.0164) | (0.0196) | (0.0148) | (0.0183) |
| Post period | 0.0019 | 0.0959^**^ | 0.1322^**^ | 0.0194 | 0.0790^*^ |
|  | (0.0337) | (0.0350) | (0.0415) | (0.0305) | (0.0383) |
| Age | 0.0003 | 0.0008 | 0.0065 | –0.0016 | 0.0087 |
|  | (0.0102) | (0.0099) | (0.0118) | (0.0093) | (0.0109) |
| *Education (reference: Less than high school)* | | | | | |
| High school diploma | –0.0056 | 0.0057 | 0.0218 | 0.0091 | 0.0303 |
|  | (0.0219) | (0.0226) | (0.0269) | (0.0216) | (0.0262) |
| College or higher | 0.1205^**^ | 0.0248 | 0.1394^*^ | –0.0417 | 0.0124 |
|  | (0.0450) | (0.0414) | (0.0590) | (0.0392) | (0.0511) |
| Married | 0.0177 | –0.0204 | –0.0002 | –0.0022 | –0.0013 |
|  | (0.0182) | (0.0204) | (0.0242) | (0.0169) | (0.0215) |
| *Rurality of residence location (reference: Urban)* | | | | | |
| Large rural | 0.0070 | 0.1402^**^ | 0.1560^**^ | 0.0808^*^ | 0.1121^*^ |
|  | (0.0438) | (0.0461) | (0.0540) | (0.0390) | (0.0493) |
| Small rural | 0.0194 | –0.0049 | –0.0190 | 0.0061 | 0.0517 |
|  | (0.0465) | (0.0489) | (0.0573) | (0.0414) | (0.0523) |
| BMI | 0.0053 | 0.1483^**^ | 0.1679^**^ | 0.0767 | 0.1282^*^ |
|  | (0.0480) | (0.0548) | (0.0607) | (0.0469) | (0.0559) |
| Smoking | 0.0574 | 0.0108 | –0.0078 | 0.0027 | 0.0788 |
|  | (0.0540) | (0.0678) | (0.0774) | (0.0537) | (0.0598) |
| Plurality | –0.0000 | 0.0002 | 0.0029 | –0.0037 | –0.0024 |
|  | (0.0026) | (0.0025) | (0.0030) | (0.0020) | (0.0026) |

^a^Results reported in this table coefficients from the fully-specified difference-in-differences model. Fixed-effects linear probability models were estimated. All models included linear time trend and month dummies. Standard errors in parentheses are adjusted for intraclass correlation.

^*^*p*<.05, ^**^*p*<.01.

**Appendix Table 3. Effect of ACA Medicaid expansion on psychotherapy for depression among perinatal women in Oregon Medicaid: coefficients^a^**

|  | Perinatal Period: | | | | |
| --- | --- | --- | --- | --- | --- |
|  | First trimester | Second trimester | Third trimester | Two months postpartum | Six months postpartum |
| Policy groupXpost period | –0.0250 | –0.0078 | –0.0370 | 0.0129 | 0.0328^*^ |
|  | (0.0149) | (0.0155) | (0.0191) | (0.0120) | (0.0149) |
| Post period | 0.0214 | 0.0599 | 0.1466^***^ | –0.0169 | 0.0982^**^ |
|  | (0.0313) | (0.0327) | (0.0402) | (0.0253) | (0.0314) |
| Age | –0.0045 | 0.0015 | 0.0062 | 0.0004 | 0.0075 |
|  | (0.0103) | (0.0107) | (0.0132) | (0.0083) | (0.0103) |
| *Education (reference: Less than high school)* | | | | | |
| High school diploma | –0.0062 | 0.0107 | 0.0329 | 0.0103 | 0.0028 |
|  | (0.0209) | (0.0219) | (0.0269) | (0.0169) | (0.0210) |
| College or higher | 0.0653 | 0.0215 | 0.1161^*^ | –0.0404 | –0.0243 |
|  | (0.0410) | (0.0428) | (0.0526) | (0.0331) | (0.0411) |
| Married | 0.0016 | –0.0028 | 0.0214 | 0.0119 | 0.0146 |
|  | (0.0171) | (0.0178) | (0.0219) | (0.0138) | (0.0171) |
| *Rurality of residence location (reference: Urban)* | | | | | |
| Large rural | 0.0088 | 0.1058^*^ | 0.1245^*^ | 0.0270 | 0.0551 |
|  | (0.0439) | (0.0458) | (0.0564) | (0.0355) | (0.0441) |
| Small rural | 0.0691 | 0.0437 | –0.0011 | –0.0286 | 0.0506 |
|  | (0.0466) | (0.0487) | (0.0599) | (0.0377) | (0.0468) |
| BMI | –0.0004 | 0.0019 | 0.0027 | –0.0025 | –0.0019 |
|  | (0.0021) | (0.0022) | (0.0027) | (0.0017) | (0.0021) |
| Smoking | 0.0022 | –0.0416^*^ | –0.0035 | –0.0116 | –0.0302 |
|  | (0.0201) | (0.0210) | (0.0258) | (0.0162) | (0.0201) |
| Plurality | 0.1633^**^ | 0.1339^*^ | 0.0248 | 0.0317 | 0.0166 |
|  | (0.0498) | (0.0520) | (0.0640) | (0.0403) | (0.0500) |

^a^Results reported in this table coefficients from the fully-specified difference-in-differences model. Fixed-effects linear probability models were estimated. All models included linear time trend and month dummies. Standard errors in parentheses are adjusted for intraclass correlation.

^*^*p*<.05, ^**^*p*<.01, ^***^*p*<.001.

**Appendix Table 4. Effect of ACA Medicaid expansion on pharmacotherapy (antidepressants) for depression among perinatal women in Oregon Medicaid: coefficients^a^**

|  | Perinatal Period: | | | | |
| --- | --- | --- | --- | --- | --- |
|  | First trimester | Second trimester | Third trimester | Two months postpartum | Six months postpartum |
| Policy groupXpost period | 0.0230^**^ | 0.0134 | 0.0082 | 0.0075 | –0.0014 |
|  | (0.0083) | (0.0080) | (0.0077) | (0.0114) | (0.0143) |
| Post period | –0.0076 | –0.0268 | –0.0007 | –0.0170 | –0.1055^***^ |
|  | (0.0175) | (0.0169) | (0.0163) | (0.0241) | (0.0301) |
| Age | –0.0078 | –0.0045 | –0.0013 | 0.0101 | 0.0220^*^ |
|  | (0.0057) | (0.0055) | (0.0053) | (0.0079) | (0.0099) |
| *Education (reference: Less than high school)* | | | | | |
| High school diploma | –0.0039 | –0.0116 | –0.0084 | 0.0069 | 0.0156 |
|  | (0.0117) | (0.0113) | (0.0109) | (0.0161) | (0.0201) |
| College or higher | 0.0652^**^ | –0.0027 | –0.0378 | 0.0037 | –0.0245 |
|  | (0.0229) | (0.0221) | (0.0213) | (0.0315) | (0.0394) |
| Married | 0.0022 | 0.0051 | –0.0012 | –0.0052 | 0.0093 |
|  | (0.0095) | (0.0092) | (0.0089) | (0.0131) | (0.0164) |
| *Rurality of residence location (reference: Urban)* | | | | | |
| Large rural | 0.0134 | 0.0180 | 0.0168 | –0.0070 | –0.0540 |
|  | (0.0245) | (0.0237) | (0.0229) | (0.0338) | (0.0422) |
| Small rural | 0.0083 | –0.0239 | –0.0296 | –0.0442 | –0.0622 |
|  | (0.0260) | (0.0251) | (0.0243) | (0.0359) | (0.0449) |
| BMI | 0.0025^*^ | –0.0003 | –0.0022 | 0.0011 | 0.0047^*^ |
|  | (0.0012) | (0.0011) | (0.0011) | (0.0016) | (0.0020) |
| Smoking | –0.0255^*^ | 0.0131 | 0.0121 | 0.0102 | –0.0098 |
|  | (0.0112) | (0.0108) | (0.0105) | (0.0154) | (0.0193) |
| Plurality | –0.0178 | –0.0018 | –0.0242 | –0.0084 | 0.0532 |
|  | (0.0278) | (0.0269) | (0.0260) | (0.0383) | (0.0479) |

^a^Results reported in this table coefficients from the fully-specified difference-in-differences model. Fixed-effects linear probability models were estimated. All models included linear time trend and month dummies. Standard errors in parentheses are adjusted for intraclass correlation.

^*^*p*<.05, ^**^*p*<.01, ^***^*p*<.001.

**Appendix Table 5. Effect of ACA Medicaid expansion on combined treatment (psychotherapy + antidepressants) for depression among perinatal women in Oregon Medicaid: coefficients^a^**

|  | Perinatal Period: | | | | |
| --- | --- | --- | --- | --- | --- |
|  | First trimester | Second trimester | Third trimester | Two months postpartum | Six months postpartum |
| Policy groupXpost period | 0.0098^*^ | 0.0034 | 0.0084 | 0.0112 | 0.0183 |
|  | (0.0050) | (0.0049) | (0.0059) | (0.0060) | (0.0098) |
| Post period | –0.0112 | 0.0127 | –0.0051 | 0.0212 | –0.0300 |
|  | (0.0099) | (0.0111) | (0.0116) | (0.0123) | (0.0212) |
| Age | –0.0002 | –0.0009 | 0.0024 | –0.0021 | –0.0076 |
|  | (0.0020) | (0.0018) | (0.0024) | (0.0025) | (0.0045) |
| *Education (reference: Less than high school)* | | | | | |
| High school diploma | –0.0055 | 0.0019 | –0.0110 | 0.0011 | 0.0013 |
|  | (0.0049) | (0.0047) | (0.0062) | (0.0085) | (0.0140) |
| College or higher | 0.0144 | 0.0117 | 0.0309 | 0.0133 | 0.0316 |
|  | (0.0137) | (0.0157) | (0.0225) | (0.0216) | (0.0293) |
| Married | 0.0067 | –0.0084 | –0.0177^*^ | –0.0082 | –0.0124 |
|  | (0.0069) | (0.0061) | (0.0069) | (0.0066) | (0.0115) |
| *Rurality of residence location (reference: Urban)* | | | | | |
| Large rural | 0.0205 | 0.0268 | 0.0109 | 0.0199 | 0.0489 |
|  | (0.0253) | (0.0238) | (0.0267) | (0.0176) | (0.0354) |
| Small rural | –0.0024 | –0.0157 | 0.0043 | 0.0309 | 0.0340 |
|  | (0.0235) | (0.0151) | (0.0272) | (0.0235) | (0.0353) |
| BMI | 0.0013 | –0.0005 | 0.0001 | –0.0003 | –0.0010 |
|  | (0.0007) | (0.0008) | (0.0008) | (0.0009) | (0.0014) |
| Smoking | –0.0022 | 0.0168 | 0.0068 | 0.0021 | 0.0294 |
|  | (0.0108) | (0.0095) | (0.0114) | (0.0111) | (0.0181) |
| Plurality | –0.0170 | –0.0174 | 0.0312 | –0.0022 | –0.0035 |
|  | (0.0266) | (0.0151) | (0.0223) | (0.0028) | (0.0305) |

^a^Results reported in this table coefficients from the fully-specified difference-in-differences model. Fixed-effects linear probability models were estimated. All models included linear time trend and month dummies. Standard errors in parentheses are adjusted for intraclass correlation.

^*^*p*<.05.

**Appendix Table 6. The effect of ACA Medicaid expansion on depression screening among perinatal women in Oregon Medicaid by race/ethnicity: coefficients^a^**

|  | Perinatal Period: | | | | |
| --- | --- | --- | --- | --- | --- |
|  | First trimester | Second trimester | Third trimester | Two–month postpartum | Six–month postpartum |
| PolicyXPost | 0.0031 | –0.0115 | –0.0242 | 0.0206 | 0.0358 |
|  | (0.0183) | (0.0188) | (0.0224) | (0.0163) | (0.0206) |
| PolicyXpost XBlack | 0.0368 | –0.0417 | 0.0042 | –0.0188 | –0.0622 |
|  | (0.0645) | (0.0661) | (0.0788) | (0.0574) | (0.0726) |
| PolicyXpost  XAIAN^b^ | –0.0123 | –0.0339 | –0.0699 | 0.0245 | 0.1017 |
|  | (0.0497) | (0.0509) | (0.0607) | (0.0442) | (0.0559) |
| PolicyXpost XAsian | –0.0738 | –0.0105 | –0.1175 | 0.0284 | 0.0020 |
|  | (0.0870) | (0.0892) | (0.1063) | (0.0775) | (0.0979) |
| PolicyXpost XNHPI^c^ | –0.0232 | –0.0812 | –0.0193 | –0.0441 | –0.0861 |
|  | (0.1114) | (0.1143) | (0.1361) | (0.0992) | (0.1253) |
| PolicyXpost XHispanic | –0.0160 | 0.0131 | 0.0001 | –0.0005 | –0.0165 |
|  | (0.0323) | (0.0331) | (0.0395) | (0.0288) | (0.0364) |
| Post period | 0.0012 | 0.0944^**^ | 0.1325^**^ | 0.0212 | 0.0827^*^ |
|  | (0.0340) | (0.0349) | (0.0416) | (0.0303) | (0.0383) |
| Age | 0.0000 | 0.0007 | 0.0061 | –0.0018 | 0.0087 |
|  | (0.0112) | (0.0114) | (0.0136) | (0.0099) | (0.0126) |
| *Education (reference: Less than high school)* | | | | | |
| High school diploma | –0.0054 | 0.0049 | 0.0208 | 0.0092 | 0.0312 |
|  | (0.0228) | (0.0234) | (0.0278) | (0.0203) | (0.0256) |
| College or higher | 0.1207^**^ | 0.0238 | 0.1387^*^ | –0.0416 | 0.0145 |
|  | (0.0445) | (0.0457) | (0.0544) | (0.0397) | (0.0501) |
| Married | 0.0182 | –0.0202 | –0.0000 | –0.0022 | –0.0012 |
|  | (0.0186) | (0.0191) | (0.0227) | (0.0165) | (0.0209) |
| *Rurality of residence location (reference: Urban)* | | | | | |
| Large rural | 0.0049 | 0.1480^**^ | 0.1662^**^ | 0.0770 | 0.1298^*^ |
|  | (0.0478) | (0.0490) | (0.0583) | (0.0425) | (0.0537) |
| Small rural | 0.0580 | 0.0090 | –0.0098 | 0.0031 | 0.0817 |
|  | (0.0508) | (0.0521) | (0.0620) | (0.0452) | (0.0571) |
| BMI | –0.0001 | 0.0003 | 0.0028 | –0.0036 | –0.0023 |
|  | (0.0023) | (0.0024) | (0.0028) | (0.0021) | (0.0026) |
| Smoking | –0.0149 | –0.0451^*^ | 0.0048 | –0.0241 | 0.0144 |
|  | (0.0218) | (0.0224) | (0.0267) | (0.0195) | (0.0246) |
| Plurality | 0.1585^**^ | 0.0864 | 0.0582 | 0.0213 | 0.0227 |
|  | (0.0543) | (0.0557) | (0.0663) | (0.0483) | (0.0611) |

^a^Reported are coefficients from the fully-specified difference-in-differences model that included additional triple interaction terms of the policy group indicator, post-ACA period indicator and race/ethnicity category. Fixed-effects linear probability models were estimated. All models included linear time trend and month dummies. Standard errors in parentheses are adjusted for intraclass correlation.

^b^AIAN=American Indian/Alaskan Native.

^c^NHPI=Native Hawaiian/Pacific Islander.

^*^*p*<.05, ^**^*p*<.01.

**Appendix Table 7. The effect of ACA Medicaid expansion on psychotherapy among perinatal women in Oregon Medicaid by race/ethnicity: coefficients^a^**

|  | Perinatal Period: | | | | |
| --- | --- | --- | --- | --- | --- |
|  | First trimester | Second trimester | Third trimester | Two–month postpartum | Six–month postpartum |
| PolicyXPost | –0.0283 | –0.0078 | –0.0352 | 0.0138 | 0.0354^*^ |
|  | (0.0169) | (0.0176) | (0.0216) | (0.0136) | (0.0169) |
| PolicyXpost XBlack | (0.0313) | (0.0327) | (0.0402) | (0.0254) | (0.0314) |
|  | 0.0328 | –0.0368 | –0.0193 | –0.0164 | –0.0314 |
| PolicyXpost  XAIAN^b^ | (0.0594) | (0.0620) | (0.0762) | (0.0480) | (0.0596) |
|  | 0.0078 | –0.0191 | –0.0426 | 0.0044 | 0.0328 |
| PolicyXpost XAsian | (0.0457) | (0.0477) | (0.0587) | (0.0370) | (0.0459) |
|  | –0.0593 | –0.0032 | –0.0989 | 0.0148 | –0.0214 |
| PolicyXpost XNHPI^c^ | (0.0801) | (0.0836) | (0.1028) | (0.0648) | (0.0803) |
|  | 0.0126 | –0.0819 | –0.0021 | 0.0412 | 0.0809 |
| PolicyXpost XHispanic | (0.1025) | (0.1071) | (0.1316) | (0.0829) | (0.1029) |
|  | 0.0139 | 0.0206 | 0.0181 | –0.0074 | –0.0228 |
| Post period | (0.0297) | (0.0311) | (0.0382) | (0.0241) | (0.0299) |
|  | 0.0215 | 0.0600 | 0.1479^***^ | –0.0167 | 0.0992^**^ |
| Age | –0.0048 | 0.0012 | 0.0058 | 0.0007 | 0.0079 |
|  | (0.0103) | (0.0107) | (0.0132) | (0.0083) | (0.0103) |
| *Education (reference: Less than high school)* | | | | | |
| High school diploma | –0.0067 | 0.0097 | 0.0316 | 0.0106 | 0.0037 |
|  | (0.0210) | (0.0219) | (0.0269) | (0.0170) | (0.0211) |
| College or higher | 0.0659 | 0.0206 | 0.1159^*^ | –0.0401 | –0.0227 |
|  | (0.0410) | (0.0428) | (0.0526) | (0.0332) | (0.0411) |
| Married | 0.0016 | –0.0027 | 0.0212 | 0.0117 | 0.0142 |
|  | (0.0171) | (0.0179) | (0.0220) | (0.0138) | (0.0172) |
| *Rurality of residence location (reference: Urban)* | | | | | |
| Large rural | 0.0090 | 0.1056^*^ | 0.1235^*^ | 0.0270 | 0.0555 |
|  | (0.0440) | (0.0459) | (0.0564) | (0.0356) | (0.0441) |
| Small rural | 0.0683 | 0.0417 | –0.0036 | –0.0280 | 0.0527 |
|  | (0.0467) | (0.0488) | (0.0600) | (0.0378) | (0.0469) |
| BMI | –0.0004 | 0.0020 | 0.0026 | –0.0025 | –0.0018 |
|  | (0.0021) | (0.0022) | (0.0027) | (0.0017) | (0.0021) |
| Smoking | 0.0022 | –0.0417^*^ | –0.0043 | –0.0117 | –0.0303 |
|  | (0.0201) | (0.0210) | (0.0258) | (0.0163) | (0.0202) |
| Plurality | 0.1614^**^ | 0.1355^**^ | 0.0260 | 0.0325 | 0.0170 |
|  | (0.0499) | (0.0521) | (0.0641) | (0.0404) | (0.0501) |

^a^Reported are coefficients from the fully-specified difference-in-differences model that included additional triple interaction terms of the policy group indicator, post-ACA period indicator and race/ethnicity category. Fixed-effects linear probability models were estimated. All models included linear time trend and month dummies. Standard errors in parentheses are adjusted for intraclass correlation.

^b^AIAN=American Indian/Alaskan Native.

^c^NHPI=Native Hawaiian/Pacific Islander.

^*^*p*<.05, ^**^*p*<.01, ^***^*p*<.001.

**Appendix Table 8. The effect of ACA Medicaid expansion on pharmacotherapy among perinatal women in Oregon Medicaid by race/ethnicity: coefficients^a^**

|  | Perinatal Period: | | | | |
| --- | --- | --- | --- | --- | --- |
|  | First trimester | Second trimester | Third trimester | Two–month postpartum | Six–month postpartum |
| PolicyXPost | 0.0305^**^ | 0.0191^*^ | 0.0157 | –0.0028 | –0.0132 |
|  | (0.0094) | (0.0091) | (0.0087) | (0.0130) | (0.0162) |
| PolicyXpost XBlack | –0.0350 | –0.0217 | –0.0607^*^ | 0.0304 | 0.0302 |
|  | (0.0331) | (0.0320) | (0.0308) | (0.0456) | (0.0571) |
| PolicyXpost  XAIAN^b^ | –0.0217 | 0.0109 | 0.0142 | 0.1169^***^ | 0.0989^*^ |
|  | (0.0255) | (0.0246) | (0.0237) | (0.0351) | (0.0439) |
| PolicyXpost XAsian | 0.0154 | 0.0032 | –0.0202 | 0.0321 | 0.0830 |
|  | (0.0447) | (0.0431) | (0.0415) | (0.0615) | (0.0769) |
| PolicyXpost XNHPI^c^ | –0.0361 | –0.0227 | –0.0227 | –0.0206 | 0.0943 |
|  | (0.0572) | (0.0552) | (0.0532) | (0.0788) | (0.0986) |
| PolicyXpost XHispanic | –0.0240 | –0.0286 | –0.0260 | 0.0051 | 0.0054 |
|  | (0.0166) | (0.0160) | (0.0154) | (0.0228) | (0.0286) |
| Post period | –0.0080 | –0.0267 | –0.0014 | –0.0152 | –0.1040^***^ |
|  | (0.0175) | (0.0169) | (0.0163) | (0.0241) | (0.0301) |
| Age | –0.0076 | –0.0043 | –0.0009 | 0.0099 | 0.0223^*^ |
|  | (0.0057) | (0.0055) | (0.0053) | (0.0079) | (0.0099) |
| *Education (reference: Less than high school)* | | | | | |
| High school diploma | –0.0033 | –0.0108 | –0.0076 | 0.0077 | 0.0167 |
|  | (0.0117) | (0.0113) | (0.0109) | (0.0161) | (0.0202) |
| College or higher | 0.0646^**^ | –0.0023 | –0.0370 | 0.0059 | –0.0226 |
|  | (0.0229) | (0.0221) | (0.0213) | (0.0315) | (0.0394) |
| Married | 0.0024 | 0.0053 | –0.0011 | –0.0051 | 0.0087 |
|  | (0.0095) | (0.0092) | (0.0089) | (0.0131) | (0.0164) |
| *Rurality of residence location (reference: Urban)* | | | | | |
| Large rural | 0.0128 | 0.0180 | 0.0171 | –0.0046 | –0.0520 |
|  | (0.0245) | (0.0237) | (0.0228) | (0.0338) | (0.0422) |
| Small rural | 0.0092 | –0.0220 | –0.0278 | –0.0418 | –0.0598 |
|  | (0.0261) | (0.0252) | (0.0242) | (0.0359) | (0.0449) |
| BMI | 0.0025^*^ | –0.0002 | –0.0020 | 0.0012 | 0.0047^*^ |
|  | (0.0012) | (0.0011) | (0.0011) | (0.0016) | (0.0020) |
| Smoking | –0.0254^*^ | 0.0136 | 0.0122 | 0.0117 | –0.0090 |
|  | (0.0112) | (0.0108) | (0.0104) | (0.0154) | (0.0193) |
| Plurality | –0.0156 | –0.0011 | –0.0220 | –0.0130 | 0.0496 |
|  | (0.0279) | (0.0269) | (0.0259) | (0.0384) | (0.0480) |

^a^Reported are coefficients from the fully-specified difference-in-differences model that included additional triple interaction terms of the policy group indicator, post-ACA period indicator and race/ethnicity category. Fixed-effects linear probability models were estimated. All models included linear time trend and month dummies. Standard errors in parentheses are adjusted for intraclass correlation.

^b^AIAN=American Indian/Alaskan Native.

^c^NHPI=Native Hawaiian/Pacific Islander.

^*^*p*<.05, ^**^*p*<.01, ^***^*p*<.001.

**Appendix Table 9. The effect of ACA Medicaid expansion on combined treatment among perinatal women in Oregon Medicaid by race/ethnicity: coefficients^a^**

|  | Perinatal Period: | | | | |
| --- | --- | --- | --- | --- | --- |
|  | First trimester | Second trimester | Third trimester | Two–month postpartum | Six–month postpartum |
| PolicyXPost | 0.0111 | 0.0033 | 0.0120 | 0.0143^*^ | 0.0234^*^ |
|  | (0.0059) | (0.0054) | (0.0066) | (0.0066) | (0.0113) |
| PolicyXpost XBlack | –0.0123 | –0.0058 | 0.0026 | –0.0152 | –0.0093 |
|  | (0.0207) | (0.0191) | (0.0234) | (0.0234) | (0.0398) |
| PolicyXpost  XAIAN^b^ | 0.0293 | –0.0045 | –0.0034 | –0.0119 | –0.0200 |
|  | (0.0160) | (0.0147) | (0.0180) | (0.0180) | (0.0307) |
| PolicyXpost XAsian | –0.0150 | –0.0066 | –0.0177 | –0.0169 | –0.0093 |
|  | (0.0279) | (0.0258) | (0.0315) | (0.0316) | (0.0537) |
| PolicyXpost XNHPI^c^ | –0.0156 | –0.0087 | –0.0134 | –0.0152 | –0.0386 |
|  | (0.0358) | (0.0330) | (0.0404) | (0.0404) | (0.0688) |
| PolicyXpost XHispanic | –0.0118 | 0.0067 | –0.0154 | –0.0061 | –0.0145 |
|  | (0.0104) | (0.0096) | (0.0117) | (0.0117) | (0.0200) |
| Post period | –0.0106 | 0.0114 | –0.0053 | 0.0211 | –0.0305 |
|  | (0.0109) | (0.0101) | (0.0123) | (0.0124) | (0.0210) |
| Age | –0.0001 | –0.0009 | 0.0024 | –0.0021 | –0.0076 |
|  | (0.0036) | (0.0033) | (0.0040) | (0.0040) | (0.0069) |
| *Education (reference: Less than high school)* | | | | | |
| High school diploma | –0.0050 | 0.0017 | –0.0106 | 0.0011 | 0.0015 |
|  | (0.0073) | (0.0067) | (0.0083) | (0.0083) | (0.0141) |
| College or higher | 0.0153 | 0.0116 | 0.0310 | 0.0132 | 0.0311 |
|  | (0.0143) | (0.0132) | (0.0162) | (0.0162) | (0.0275) |
| Married | 0.0069 | –0.0084 | –0.0174^**^ | –0.0081 | –0.0121 |
|  | (0.0060) | (0.0055) | (0.0067) | (0.0067) | (0.0115) |
| *Rurality of residence location (reference: Urban)* | | | | | |
| Large rural | 0.0209 | 0.0270 | 0.0107 | 0.0196 | 0.0484 |
|  | (0.0153) | (0.0141) | (0.0173) | (0.0173) | (0.0295) |
| Small rural | –0.0011 | –0.0162 | 0.0051 | 0.0309 | 0.0344 |
|  | (0.0163) | (0.0150) | (0.0184) | (0.0184) | (0.0314) |
| BMI | 0.0013 | –0.0004 | 0.0001 | –0.0003 | –0.0010 |
|  | (0.0007) | (0.0007) | (0.0008) | (0.0008) | (0.0014) |
| Smoking | –0.0018 | 0.0166^*^ | 0.0070 | 0.0020 | 0.0295^*^ |
|  | (0.0070) | (0.0065) | (0.0079) | (0.0079) | (0.0135) |
| Plurality | –0.0174 | –0.0170 | 0.0312 | –0.0013 | –0.0025 |
|  | (0.0174) | (0.0161) | (0.0197) | (0.0197) | (0.0335) |

^a^Reported are coefficients from the fully-specified difference-in-differences model that included additional triple interaction terms of the policy group indicator, post-ACA period indicator and race/ethnicity category. Fixed-effects linear probability models were estimated. All models included linear time trend and month dummies. Standard errors in parentheses are adjusted for intraclass correlation.

^b^AIAN=American Indian/Alaskan Native.

^c^NHPI=Native Hawaiian/Pacific Islander.

^*^*p*<.05, ^**^*p*<.01.

**Appendix Table 10. The effect of ACA Medicaid expansion on depression screening among perinatal women in Oregon Medicaid by rurality: coefficients^a^**

|  | Perinatal Period: | | | | | | | | | |
| --- | --- | --- | --- | --- | --- | --- | --- | --- | --- | --- |
|  | First trimester | | Second trimester | | Third trimester | | Two–month postpartum | | Six–month postpartum | |
| PolicyXPost | | –0.0054 | | –0.0168 | | –0.0246 | | 0.0299^*^ | | 0.0455^*^ |
|  |  | (0.0171) | | (0.0176) | | (0.0209) | | (0.0152) | | (0.0193) |
| PolicyXpost XLarge rural | | 0.0541 | | 0.0075 | | –0.0246 | | –0.0018 | | –0.0123 |
|  |  | (0.0404) | | (0.0415) | | (0.0495) | | (0.0360) | | (0.0455) |
| PolicyXpost  XSmall rural | | –0.0348 | | 0.0218 | | –0.0666 | | –0.1461^**^ | | –0.1438^*^ |
|  |  | (0.0575) | | (0.0590) | | (0.0703) | | (0.0512) | | (0.0648) |
| Post period | | 0.0010 | | 0.0959^**^ | | 0.1322^**^ | | 0.0188 | | 0.0786^*^ |
|  | | (0.0340) | | (0.0349) | | (0.0416) | | (0.0303) | | (0.0383) |
| Age | | 0.0000 | | 0.0009 | | 0.0064 | | –0.0020 | | 0.0083 |
|  | | (0.0111) | | (0.0114) | | (0.0136) | | (0.0099) | | (0.0126) |
| *Education (reference: Less than high school)* | | | | | | | | | | |
| High school diploma | | –0.0054 | | 0.0057 | | 0.0216 | | 0.0089 | | 0.0300 |
|  |  | (0.0228) | | (0.0234) | | (0.0278) | | (0.0203) | | (0.0256) |
| College or higher | | 0.1220^**^ | | 0.0250 | | 0.1386^*^ | | –0.0421 | | 0.0117 |
|  |  | (0.0445) | | (0.0457) | | (0.0544) | | (0.0396) | | (0.0501) |
| Married | | 0.0173 | | –0.0206 | | 0.0004 | | –0.0014 | | –0.0005 |
|  | | (0.0186) | | (0.0191) | | (0.0227) | | (0.0165) | | (0.0209) |
| *Rurality of residence location (reference: Urban)* | | | | | | | | | | |
| Large rural | | –0.0064 | | 0.1463^**^ | | 0.1744^**^ | | 0.0792 | | 0.1331^*^ |
|  |  | (0.0486) | | (0.0499) | | (0.0594) | | (0.0433) | | (0.0547) |
| Small rural | | 0.0672 | | 0.0055 | | 0.0082 | | 0.0388 | | 0.1141 |
|  |  | (0.0526) | | (0.0540) | | (0.0643) | | (0.0469) | | (0.0593) |
| BMI | | 0.0001 | | 0.0002 | | 0.0029 | | –0.0036 | | –0.0024 |
|  | | (0.0023) | | (0.0024) | | (0.0028) | | (0.0021) | | (0.0026) |
| Smoking | | –0.0148 | | –0.0450^*^ | | 0.0064 | | –0.0229 | | 0.0146 |
|  | | (0.0218) | | (0.0224) | | (0.0267) | | (0.0194) | | (0.0246) |
| Plurality | | 0.1616^**^ | | 0.0839 | | 0.0571 | | 0.0230 | | 0.0249 |
|  | | (0.0542) | | (0.0556) | | (0.0662) | | (0.0482) | | (0.0610) |
| Parity | | –0.0064 | | 0.1463^**^ | | 0.1744^**^ | | 0.0792 | | 0.1331^*^ |
|  | | (0.0486) | | (0.0499) | | (0.0594) | | (0.0433) | | (0.0547) |

^a^Reported are coefficients from the fully-specified difference-in-differences model that included additional triple interaction terms of the policy group indicator, post-ACA period indicator, and rural/urban category. Fixed-effects linear probability models were estimated. All models included linear time trend and month dummies. Standard errors in parentheses are adjusted for intraclass correlation.

^*^*p*<.05, ^**^*p*<.01.

**Appendix Table 11. The effect of ACA Medicaid expansion on psychotherapy among perinatal women in Oregon Medicaid by rurality: coefficients^a^**

|  | Perinatal Period: | | | | | | | | | |
| --- | --- | --- | --- | --- | --- | --- | --- | --- | --- | --- |
|  | First trimester | | Second trimester | | Third trimester | | Two–month postpartum | | Six–month postpartum | |
| PolicyXPost | | –0.0262 | | –0.0055 | | –0.0339 | | 0.0336^*^ | | 0.0615^***^ |
|  |  | (0.0158) | | (0.0165) | | (0.0202) | | (0.0138) | | (0.0179) |
| PolicyXpost XLarge rural | | 0.0237 | | –0.0144 | | –0.0161 | | –0.0155 | | –0.0156 |
|  |  | (0.0372) | | (0.0389) | | (0.0478) | | (0.0326) | | (0.0423) |
| PolicyXpost  XSmall rural | | –0.0289 | | –0.0139 | | –0.0255 | | –0.1446^**^ | | –0.1616^**^ |
|  |  | (0.0529) | | (0.0553) | | (0.0680) | | (0.0464) | | (0.0602) |
| Post period | | 0.0210 | | 0.0601 | | 0.1468^***^ | | 0.0039 | | 0.0677 |
|  | | (0.0313) | | (0.0327) | | (0.0402) | | (0.0274) | | (0.0356) |
| Age | | –0.0046 | | 0.0014 | | 0.0062 | | –0.0021 | | –0.0005 |
|  | | (0.0103) | | (0.0107) | | (0.0132) | | (0.0090) | | (0.0117) |
| *Education (reference: Less than high school)* | | | | | | | | | | |
| High school diploma | | –0.0062 | | 0.0106 | | 0.0328 | | 0.0112 | | 0.0039 |
|  |  | (0.0209) | | (0.0219) | | (0.0269) | | (0.0184) | | (0.0238) |
| College or higher | | 0.0659 | | 0.0210 | | 0.1156^*^ | | –0.0279 | | 0.0065 |
|  |  | (0.0410) | | (0.0428) | | (0.0526) | | (0.0359) | | (0.0466) |
| Married | | 0.0016 | | –0.0026 | | 0.0217 | | 0.0047 | | 0.0032 |
|  | | (0.0171) | | (0.0178) | | (0.0219) | | (0.0150) | | (0.0194) |
| *Rurality of residence location (reference: Urban)* | | | | | | | | | | |
| Large rural | | 0.0039 | | 0.1093^*^ | | 0.1285^*^ | | 0.0526 | | 0.1099^*^ |
|  |  | (0.0447) | | (0.0467) | | (0.0575) | | (0.0392) | | (0.0508) |
| Small rural | | 0.0767 | | 0.0468 | | 0.0049 | | 0.0376 | | 0.1242^*^ |
|  |  | (0.0484) | | (0.0506) | | (0.0622) | | (0.0424) | | (0.0550) |
| BMI | | –0.0003 | | 0.0019 | | 0.0027 | | –0.0027 | | –0.0028 |
|  | | (0.0021) | | (0.0022) | | (0.0027) | | (0.0019) | | (0.0024) |
| Smoking | | 0.0025 | | –0.0414^*^ | | –0.0031 | | –0.0077 | | 0.0012 |
|  | | (0.0201) | | (0.0210) | | (0.0258) | | (0.0176) | | (0.0228) |
| Plurality | | 0.1643^***^ | | 0.1337^*^ | | 0.0246 | | 0.0308 | | 0.0144 |
|  | | (0.0499) | | (0.0521) | | (0.0640) | | (0.0437) | | (0.0566) |
| Parity | | 0.0039 | | 0.1093^*^ | | 0.1285^*^ | | 0.0526 | | 0.1099^*^ |
|  | | (0.0447) | | (0.0467) | | (0.0575) | | (0.0392) | | (0.0508) |

^a^Reported are coefficients from the fully-specified difference-in-differences model that included additional triple interaction terms of the policy group indicator, post-ACA period indicator, and rural/urban category. Fixed-effects linear probability models were estimated. All models included linear time trend and month dummies. Standard errors in parentheses are adjusted for intraclass correlation.

^*^*p*<.05, ^**^*p*<.01, ^***^*p*<.001.

**Appendix Table 12. The effect of ACA Medicaid expansion on pharmacotherapy among perinatal women in Oregon Medicaid by rurality: coefficients^a^**

|  | Perinatal Period: | | | | | | | | | |
| --- | --- | --- | --- | --- | --- | --- | --- | --- | --- | --- |
|  | First trimester | | Second trimester | | Third trimester | | Two–month postpartum | | Six–month postpartum | |
| PolicyXPost | | 0.0216^*^ | | 0.0170^*^ | | 0.0107 | | 0.0088 | | –0.0046 |
|  |  | (0.0088) | | (0.0085) | | (0.0082) | | (0.0121) | | (0.0152) |
| PolicyXpost XLarge rural | | 0.0355 | | –0.0390 | | –0.0180 | | –0.0394 | | –0.0072 |
|  |  | (0.0208) | | (0.0201) | | (0.0194) | | (0.0286) | | (0.0358) |
| PolicyXpost  XSmall rural | | –0.0477 | | 0.0151 | | –0.0084 | | 0.0579 | | 0.0741 |
|  |  | (0.0295) | | (0.0285) | | (0.0276) | | (0.0407) | | (0.0509) |
| Post period | | –0.0083 | | –0.0262 | | –0.0005 | | –0.0162 | | –0.1051^***^ |
|  | | (0.0175) | | (0.0169) | | (0.0163) | | (0.0241) | | (0.0301) |
| Age | | –0.0080 | | –0.0044 | | –0.0013 | | 0.0103 | | 0.0222^*^ |
|  | | (0.0057) | | (0.0055) | | (0.0053) | | (0.0079) | | (0.0099) |
| *Education (reference: Less than high school)* | | | | | | | | | | |
| High school diploma | | –0.0038 | | –0.0118 | | –0.0085 | | 0.0068 | | 0.0157 |
|  |  | (0.0117) | | (0.0113) | | (0.0109) | | (0.0161) | | (0.0201) |
| College or higher | | 0.0661^**^ | | –0.0038 | | –0.0383 | | 0.0027 | | –0.0245 |
|  |  | (0.0229) | | (0.0221) | | (0.0213) | | (0.0315) | | (0.0394) |
| Married | | 0.0021 | | 0.0054 | | –0.0010 | | –0.0051 | | 0.0089 |
|  | | (0.0095) | | (0.0092) | | (0.0089) | | (0.0131) | | (0.0164) |
| *Rurality of residence location (reference: Urban)* | | | | | | | | | | |
| Large rural | | 0.0060 | | 0.0266 | | 0.0210 | | 0.0011 | | –0.0535 |
|  |  | (0.0250) | | (0.0241) | | (0.0233) | | (0.0344) | | (0.0430) |
| Small rural | | 0.0209 | | –0.0285 | | –0.0279 | | –0.0594 | | –0.0807 |
|  |  | (0.0270) | | (0.0261) | | (0.0252) | | (0.0372) | | (0.0466) |
| BMI | | 0.0026^*^ | | –0.0003 | | –0.0022^*^ | | 0.0010 | | 0.0046^*^ |
|  | | (0.0012) | | (0.0011) | | (0.0011) | | (0.0016) | | (0.0020) |
| Smoking | | –0.0250^*^ | | 0.0130 | | 0.0123 | | 0.0096 | | –0.0107 |
|  | | (0.0112) | | (0.0108) | | (0.0105) | | (0.0154) | | (0.0193) |
| Plurality | | –0.0163 | | –0.0030 | | –0.0246 | | –0.0101 | | 0.0522 |
|  | | (0.0278) | | (0.0269) | | (0.0260) | | (0.0383) | | (0.0479) |
| Parity | | 0.0060 | | 0.0266 | | 0.0210 | | 0.0011 | | –0.0535 |
|  | | (0.0250) | | (0.0241) | | (0.0233) | | (0.0344) | | (0.0430) |

^a^Reported are coefficients from the fully-specified difference-in-differences model that included additional triple interaction terms of the policy group indicator, post-ACA period indicator, and rural/urban category. Fixed-effects linear probability models were estimated. All models included linear time trend and month dummies. Standard errors in parentheses are adjusted for intraclass correlation.

^*^*p*<.05, ^**^*p*<.01, ^***^*p*<.001.

**Appendix Table 13. The effect of ACA Medicaid expansion on combined treatment among perinatal women in Oregon Medicaid by rurality: coefficients^a^**

|  | Perinatal Period: | | | | | | | | | |
| --- | --- | --- | --- | --- | --- | --- | --- | --- | --- | --- |
|  | First trimester | | Second trimester | | Third trimester | | Two–month postpartum | | Six–month postpartum | |
| PolicyXPost | | 0.0092 | | 0.0009 | | 0.0089 | | 0.0164^**^ | | 0.0248^*^ |
|  |  | (0.0055) | | (0.0051) | | (0.0062) | | (0.0062) | | (0.0106) |
| PolicyXpost XLarge rural | | 0.0020 | | 0.0208 | | 0.0109 | | –0.0261 | | –0.0266 |
|  |  | (0.0130) | | (0.0121) | | (0.0147) | | (0.0147) | | (0.0250) |
| PolicyXpost  XSmall rural | | 0.0077 | | 0.0017 | | –0.0311 | | –0.0423^*^ | | –0.0655 |
|  |  | (0.0185) | | (0.0172) | | (0.0209) | | (0.0208) | | (0.0355) |
| Post period | | –0.0112 | | 0.0124 | | –0.0054 | | 0.0214 | | –0.0299 |
|  | | (0.0109) | | (0.0102) | | (0.0123) | | (0.0123) | | (0.0210) |
| Age | | –0.0002 | | –0.0010 | | 0.0023 | | –0.0022 | | –0.0077 |
|  | | (0.0036) | | (0.0033) | | (0.0040) | | (0.0040) | | (0.0069) |
| *Education (reference: Less than high school)* | | | | | | | | | | |
| High school diploma | | –0.0055 | | 0.0020 | | –0.0110 | | 0.0010 | | 0.0011 |
|  |  | (0.0073) | | (0.0068) | | (0.0083) | | (0.0082) | | (0.0141) |
| College or higher | | 0.0145 | | 0.0123 | | 0.0311 | | 0.0124 | | 0.0307 |
|  |  | (0.0143) | | (0.0133) | | (0.0161) | | (0.0161) | | (0.0275) |
| Married | | 0.0067 | | –0.0086 | | –0.0176^**^ | | –0.0077 | | –0.0118 |
|  | | (0.0060) | | (0.0055) | | (0.0067) | | (0.0067) | | (0.0115) |
| *Rurality of residence location (reference: Urban)* | | | | | | | | | | |
| Large rural | | 0.0199 | | 0.0221 | | 0.0089 | | 0.0265 | | 0.0559 |
|  |  | (0.0156) | | (0.0145) | | (0.0176) | | (0.0176) | | (0.0300) |
| Small rural | | –0.0042 | | –0.0157 | | 0.0122 | | 0.0407^*^ | | 0.0497 |
|  |  | (0.0169) | | (0.0157) | | (0.0191) | | (0.0191) | | (0.0325) |
| BMI | | 0.0013 | | –0.0004 | | 0.0001 | | –0.0003 | | –0.0010 |
|  | | (0.0007) | | (0.0007) | | (0.0008) | | (0.0008) | | (0.0014) |
| Smoking | | –0.0023 | | 0.0167^*^ | | 0.0072 | | 0.0027 | | 0.0303^*^ |
|  | | (0.0070) | | (0.0065) | | (0.0079) | | (0.0079) | | (0.0135) |
| Plurality | | –0.0170 | | –0.0169 | | 0.0318 | | –0.0024 | | –0.0035 |
|  | | (0.0174) | | (0.0162) | | (0.0196) | | (0.0196) | | (0.0334) |
| Parity | | 0.0199 | | 0.0221 | | 0.0089 | | 0.0265 | | 0.0559 |
|  | | (0.0156) | | (0.0145) | | (0.0176) | | (0.0176) | | (0.0300) |

^a^Reported are coefficients from the fully-specified difference-in-differences model that included additional triple interaction terms of the policy group indicator, post-ACA period indicator, and rural/urban category. Fixed-effects linear probability models were estimated. All models included linear time trend and month dummies. Standard errors in parentheses are adjusted for intraclass correlation.

^*^*p*<.05, ^**^*p*<.01.

**Appendix Table 14. Comparison of the study sample to all women in Oregon Medicaid who gave live births between 2011 and 2016**

| Variable | All Oregon Medicaid women who gave live births, 2011-2016 | The sample |
| --- | --- | --- |
| Age, Mean (SD) | 28.7 (5.84) | 26.2 (5.29) |
| *Race-ethnicity* |  |  |
| White | 66.9% | 55.4% |
| Black | 2.9% | 4.1% |
| American Indian/Alaska Native | 3.1% | 4.9% |
| Asian | 5.8% | 2.3% |
| Native Hawaiian/Pacific Islander | 1.1% | 1.6% |
| Hispanic | 20.2% | 31.8% |
| *Education* |  |  |
| Less than high school | 15.0% | 27.8% |
| High school diploma | 46.9% | 59.6% |
| College and higher | 38.2% | 12.3% |
| Married | 64.2% | 49.0% |
| *Rurality* |  |  |
| Urban | 87.4% | 83.5% |
| Large rural | 9.5% | 12.3% |
| Small rural | 3.1% | 4.3% |
| Missing | 0.7% | 0.03% |
| BMI at delivery, Mean (SD) | 31.8 (6.36) | 32.4 (6.53) |
| Smoking | 12.4% | 16.9% |
| Observations | 266,600 | 7,411 |
